# How Does Load Selection and Sex Influence 1RM Prediction Using the Minimal Velocity Threshold During Free-Weight Back Squat?

**DOI:** 10.3390/sports13070224

**Published:** 2025-07-08

**Authors:** Emanuele Dello Stritto, Ruggero Romagnoli, Michele Nocchi, Maria Francesca Piacentini

**Affiliations:** 1 Department of Human Movement and Health Sciences, University of Rome “Foro Italico”, Piazza L. De Bosis 15, 00135 Rome, Italy; e.dellostritto@studenti.uniroma4.it (E.D.S.); michelenocchi07@gmail.com (M.N.); 2 Department of Theoretical and Applied Sciences, eCampus University, Via Isimbardi 10, 22060 Novedrate, Italy; ruggero.romagnoli@uniecampus.it

**Keywords:** sex difference, load–velocity relationship, velocity-based training, regression model, resistance training, injury prevention

## Abstract

The aim of the present study is to predict a 1RM through the load–velocity relationship using the minimum velocity threshold (MVT) in a free-weight back squat. Twenty-five males and twenty-five females performed a 1RM test during a free-weight back squat, based on which individual load–velocity relationships were created. Ten regression models to predict the 1RM were developed. The models included a two-point (mean propulsive velocity (MPV) ≈ 1 m·s^−1^; MPV ≈ 0.50 m·s^−1^) and a three-point linear equation (MPV ≈ 1 m·s^−1^; MPV ≈ 0.75 m·s^−1^; and MPV ≈ 0.50 m·s^−1^) with an MVT of 0.3 m·s^−1^ and 0.4 m·s^−1^ and, additionally, an MVT of 0.25 m·s^−1^ for females. The repeated measures ANOVA revealed no significant differences between the predicted and measured 1RM in males using an MVT of 0.30 m·s^−1^ and in females with an MVT of 0.25 m·s^−1^. In contrast, models using an MVT of 0.30 m·s^−1^ in females underestimated the measured 1RM, as did those using an MVT of 0.40 m·s^−1^ in both sexes. It appears possible to accurately predict the 1RM during a free-weight back squat using the load–velocity relationship. However, it is important to avoid using loads with velocities higher than 1 m·s^−1^ for the regression models and to use different MVTs for males and females.

## 1. Introduction

Resistance Training (RT) has gained significant importance over the past decades due to its substantial benefits for performance [1]. It has been demonstrated that performing RT improves both strength and endurance parameters, leading to increased power, speed, balance, and coordination in athletes [2,3]. Today, RT is recommended by major global health organizations for most populations, including adolescents, healthy adults, and the elderly [4,5,6]. The most utilized method for prescribing RT sessions is known as the “traditional method” [7,8] that utilizes the percentage of the one-repetition maximum (% 1RM) to determine training intensity. For this reason, an incremental maximal test is necessary to establish 1RM [9], a test that is time consuming and presents several challenges, such as the physical and mental fatigue experienced by athletes [10], and the increased risk of injury during subsequent training days [11,12]. In order to minimize these risks and negative aspects of the 1RM test, recent studies have explored the possibility of predicting the 1RM through sub-maximal tests [10,13,14,15,16]. These investigations have focused on using velocity as a parameter to estimate intensity, based on the inverse relationship between load and velocity, first mentioned by Hill [17].

Recent findings have generated predictive equations for the 1RM for different populations, based on the relationship found between % 1RM and barbell velocity, both with the Smith Machine and free-weights [9,10,13,14,18,19]. One of the most studied and precise methods for predicting the 1RM utilizes the Minimum Velocity Threshold (MVT), which corresponds to the barbell’s velocity at the 1RM for each specific exercise [10]. This method, commonly known as 1RM_MVT_, uses the exercise-specific MVT as the final data point in the regression model used to create the load–velocity relationship from which the 1RM is then estimated [10]. The accuracy of a 1RM prediction from the load–velocity relationship depends on the precision of these relationships [16], and it is crucial to use equations generated from individualized load–velocity relationships rather than generalized ones [20]. Additionally, it is important to consider the execution technique, which tends to vary with extremely light loads (<20%) and extremely heavy loads (>90%) [21]. Therefore, it is recommended to use loads between 20 and 90% 1RM to create the load–velocity relationship. Recent evidence suggests that using loads closer to 40% 1RM as the lightest load may lead to benefits in the accuracy of a 1RM prediction [21]. Various studies have also examined the impact of the number of loads on prediction accuracy. Using more than three loads offers no significant benefit in terms of accuracy compared to using two or three but considerably increases the test duration and fatigue [12,15].

Hence, in real training contexts, it is advised to use two or three reference loads, with these loads positioned at the two extremes of the load–velocity curve and, if used, a third point intermediate between the two extremes [22]. The study conducted by Hughes et al. [21] compared various methodologies for predicting a 1RM during a free-weight back squat, based on the load–velocity relationship: 1RM_MVT_, load at zero velocity, and the method based on the force–velocity curve [23]. The results confirmed that the most precise methodology is the 1RM_MVT_, demonstrating higher intersession reliability compared to the other tested methodologies and very strong correlations with the measured 1RM. However, despite the 1RM_MVT_ method being recognized as the most precise methodology for predicting a 1RM in a free-weight back squat, a recent review of the literature [10] concluded that it consistently overestimates the predicted 1RM compared to the actual measured value, questioning its reliability for accurate 1RM prediction. Additionally, since this method depends on MVT, and MVT has been primarily investigated in males [24], it is still questionable if and how potential differences between males and females in MVT might affect the accuracy of 1RM prediction. The few studies that have included female participants have reported inconsistent results, suggesting potential differences in MVT between males and females [24,25,26]. These differences need to be addressed to refine the 1RM_MVT_ method for broader applicability in a female population. Lastly, very recent studies have proposed the use of an “optimal” MVT, defined as the velocity that minimizes the error between the predicted and the actual measured 1RM [15,16]. However, since this value must be determined a priori through an incremental test, Chen et al. [15] recently suggested using a fixed MVT value of 0.40 m·s^−1^, which represents the average of the optimal MVTs previously observed. Nevertheless, although this optimal MVT has shown promising results in male subjects, it has never been tested in female athletes [15].

Therefore, the main aim of this research is to investigate the possibility of predicting the 1RM in the free-weight back squat using the 1RM_MVT_ methodology in both males and females by

(1) Comparing the use of two or three loads.
(2) Comparing differences between males and females.
(3) Evaluating the use of “Optimal MVT” of 0.40 m·s^−1^ in females.

We hypothesized that both the 2-point and 3-point models would successfully predict the 1RM using the aforementioned methodology. Furthermore, we hypothesized that differences between males and females in MVT would be significant and crucial for the accurate prediction of the 1RM. Lastly, we hypothesized that an MVT of 0.40 m·s^−1^ will significant underestimate the 1RM in females due to the important differences in the load–velocity relationship between males and females.

## 2. Materials and Methods

All subjects performed a maximal incremental test on the free-weight back squat. Based on the test results, the load–velocity relationship was calculated for each subject. To predict the 1RM, four models were developed for males. The four models involved a two-point linear equation (Mean Propulsive Velocity (MPV) ≈ 1 m·s^−1^; MPV ≈ 0.50 m·s^−1^) and a three-point linear equation (MPV ≈ 1 m·s^−1^; MPV ≈ 0.75 m·s^−1^; and MPV ≈ 0.50 m·s^−1^), both generated with the literature-recognized MVT for males of 0.30 m·s^−1^ and with the “optimal MVT” suggested of 0.40 m·s^−1^. The four generated equations were then used to predict the 1RM using the 1RM_MVT_ method. For the female population, the same MVTs were used. In addition, if statistically significant differences related to MVT between males and females are found, such differences will be considered in order to create two additional regression models specifically for females. Such regression models, if needed, will be constructed using the mean MVT of female participants experimentally measured during the 1RM tests.

### 2.1. Subjects

The present study included 25 males (age: 26.75 years ± 5.84; body weight: 79 kg ± 10.47; height: 179 cm ± 6.89; 6.6 ± 3.67 years of RT experience; 1RM/BW: 1.63 ± 0.32; and MVT: 0.29 ± 0.04 m·s^−1^) and 25 females (age: 24.68 years ± 3.54; body weight: 56 kg ± 4.7; height: 164 cm ± 4.66; 4.64 ± 2.89 years of RT experience; 1RM/BW: 1.57 ± 0.27; and MVT 0.25 ± 0.04 m·s^−1^). The prerequisites for participation in the study required at least two years of experience in RT with the ability to perform each repetition at the highest possible velocity during the concentric phase, an age between 18 and 35 years, and the absence of current or previous injuries. Given their prior experience with both the exercise and the execution modality, the subjects attended the laboratory on a single occasion only. After being adequately informed about the procedures, risks, and ethical aspects of the study and receiving a copy of the explanatory information sheet, subjects signed informed consent and data processing forms. The proposed study protocol was drafted in accordance with the current revision of the Helsinki Declaration, which aims to ensure the protection of the rights, integrity, and well-being of the subjects involved in experiments and was approved by CAR 75/2021/est. Participation in the study could be discontinued at any time at the request of the subject and/or staff, without the need to provide reasons and without suffering any consequences.

### 2.2. 1RM TEST

Prior to the test, subjects performed a dynamic warm-up consisting of mobility, stretching, and body weight exercises. Subjects performed a maximal incremental test on the free-weight back squat. They were instructed to perform each repetition as fast as possible. The initial load of the test was set at 20 kg for 5 repetitions. The load increased from 2.5 kg to 20 kg depending on barbell velocity (Table 1). During each repetition, verbal encouragement was provided from the researcher. The test ended when the athlete could not complete a single repetition with a given load [27]. The incremental test was recorded using a GoPro Hero 8 Black camera (GoPro, Inc., San Mateo, CA, USA) to ensure proper execution of the movement. The squat was performed to a depth where the hip crease descended below the level of the knee when viewed laterally [27]. At the end of each lift considered questionable, the footage was reviewed in slow motion by a certified FIPE (Italian weightlifting federation) referee. If the lift was deemed invalid, the participant was required to repeat the attempt with the same load before progressing to the next increment. A linear position transducer “Vitruve” (SPEED4LIFTS S.L., Madrid, Spain) was utilized to measure MPV, maximum velocity, range of motion, and power output. Recent research has demonstrated that it is reliable for low (MPV < 0.50 m·s^−1^) [28] and intermediate velocities (MPV ≤ 1 m·s^−1^) [28] when compared to other linear position transducers, considered a gold standard, and has shown one of the highest levels of reliability among all devices analyzed [28,29].

### 2.3. Sample Size Estimation and Justification

A priori power analysis was conducted to determine the required sample size for the present study. The primary analysis was the repeated measures ANOVA, involving a minimum of four within-subject comparisons. The analysis was performed using G*Power 3.1.9.7software, selecting the F test—repeated measures ANOVA, within factors, and with an a priori approach. The following parameters were set: a large partial eta-squared effect size (η^2^ = 0.20), an alpha level of 0.05, statistical power (1 − β) of 0.90, and an assumption of sphericity violation. Based on these criteria, the estimated sample size was 18 male and 18 female participants. However, considering a secondary aim of the study was to explore potential sex differences in MVT, we opted to recruit a larger sample than required for the repeated measures ANOVA. A total sample size of 25 participants per group was recruited. This decision was supported by a priori power analysis conducted using G*Power (*t* test—means: difference between two independent means, two-tailed, and α = 0.05). Assuming a large but realistic effect size (Cohen’s d = 0.80), the minimum sample size required to achieve a statistical power of 0.80 was estimated to be 25 participants per group.

### 2.4. Statistical Analysis

Data were normally distributed, according to the Kolmogorov–Smirnov test (*p* > 0.05). An unpaired samples *t*-test was conducted between the MVT of males and females. A repeated measures ANOVA was performed to compare the predicted values from various models with the actual 1RM for both males and females. Sphericity was tested using Mauchly’s test, and if found significant, the Greenhouse–Geisser correction was applied. Moreover, the validity and agreement of the predicted values with the actual 1RM for each model were investigated using Standard Error of Estimate (SEE), Coefficient of Variation (CV), Bland–Altman plots, and the Pearson correlation. SEE is calculated by taking the square root of the sum of the squared differences between the observed values and the predicted values, divided by the degrees of freedom. The CV was calculated as the ratio of the standard deviation of the predicted values from each model to their mean, both expressed as a percentage of the 1RM.

## 3. Results

Load–velocity profiles were created using MPV ≈ 1 m·s^−1^ and MPV ≈ 0.50 m·s^−1^ for the 2-point model and MPV ≈ 1 m·s^−1^; MPV ≈ 0.75 m·s^−1^; and MPV ≈ 0.50 m·s^−1^ for the 3-point model, which, according to the analysis, corresponded to 43.99 ± 6.2% 1RM, 67.86 ± 5.1% 1RM, and 86.18 ± 2.6% 1RM for males, and to 36.59 ± 7.21% 1RM, 60.34 ± 6.69% 1RM, and 79.52 ± 4.8% 1RM for females. The MVT was significantly different between males and females, T (48) = −2.652; *p* = 0.011, with an effect size of 1.00, where males showed a mean MVT of 0.29 ± 0.04 m·s^−1^, while females showed a mean MVT of 0.25 ± 0.04 m·s^−1^. Given the significant difference, two additional regression models with an MVT of 0.25 m·s^−1^ were considered for females. The Mauchly’s test of sphericity was significant (*p* < 0.05) in the repeated measures ANOVA for both the males’ and females’ regression models; therefore, the Greenhouse–Geisser correction was applied.

The repeated measures ANOVA was significant in males’ regression models, F (2.29) = 264.103; *p* < 0.001; and η^2^ = 0.917. Post hoc pairwise comparisons revealed that statistically significant differences were found between both prediction models (two and three points) constructed with the MVT of 0.4 m·s^−1^ (i.e., Optimal MVT) and the regression model with 0.3 m·s^−1^ and real 1RM; meanwhile, post hoc pairwise revealed no differences between the regression model (both two and three points) with real 1RM. Moreover, the repeated measures ANOVA was significant in females’ regression models, F (2.080) = 532.789; *p* < 0.01; and η^2^ = 0.957. Pairwise comparisons revealed that statistically significant differences were found between both the prediction models (two and three points), both constructed with the MVT of 0.3 m·s^−1^ and MVT of 0.40 m·s^−1^, and the models built with the MVT of 0.25 m·s^−1^ and the actual 1RM (*p* < 0.05). Conversely, no statistically significant differences were found between the models constructed with the MVT of 0.25 m·s^−1^ and the actual 1RM (*p* > 0.05).

**Figure 1:**
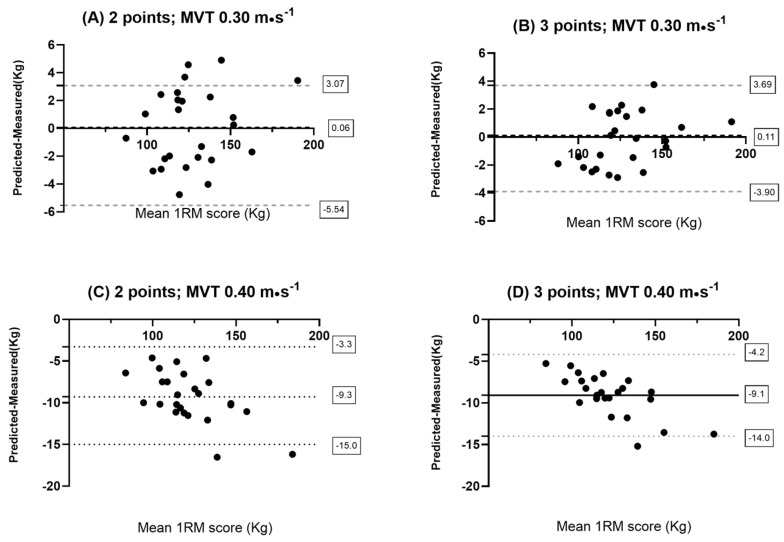
Bland–Altman plot results: (**A**) 2-point method in males with MVT of 0.3 m·s^−1^; (**B**) 3-point method in males with MVT of 0.3 m·s^−1^; (**C**) 2-point method in males with MVT of 0.4 m·s^−1^; and (**D**) 3-point method in males with MVT of 0.4 m·s^−1^.

**Figure 2:**
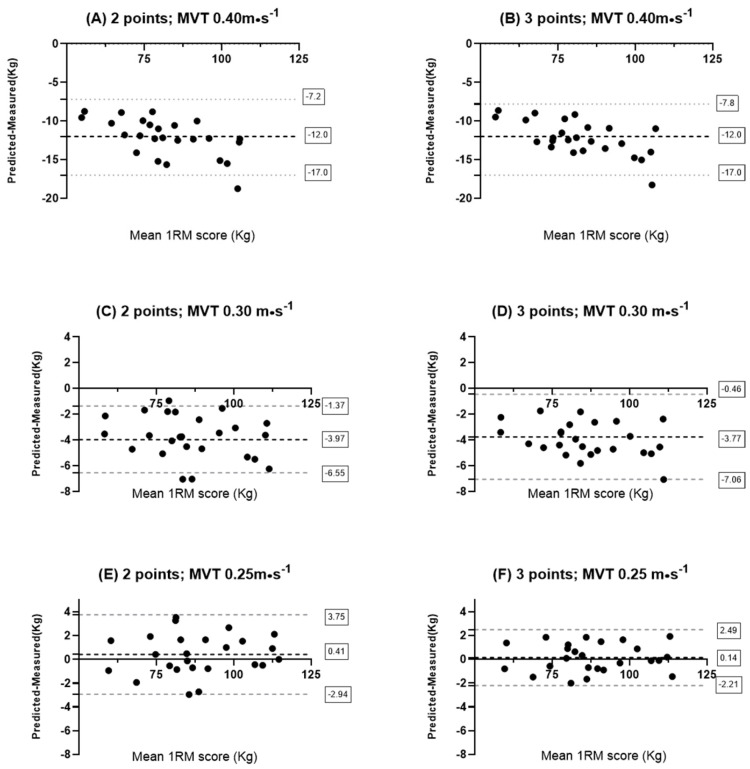
Bland–Altman plot results: (**A**) 2-point method in females with MVT of 0.4 m·s^−1^; (**B**) 3-point method in females with MVT of 0.4 m·s^−1^; (**C**) 2-point method in females with MVT of 0.3 m·s^−1^; (**D**) 3-point method in females with MVT of 0.3 m·s^−1^; (**E**) 2-point method in females with MVT of 0.25 m·s^−1^; and (**F**) 3-point method in females with MVT of 0.25 m·s^−1^.

The SEE showed similar values across all models, except for the models built with the MVT of 0.4 m·s^−1^ for both sexes and for the model built with the MVT of 0.30 m·s^−1^ in females (Table 2). The Pearson correlation (r) revealed very high values for all models (Table 2). Finally, the CV reported very low values for all models (Table 2). Lastly, the Bland–Altman plots indicated a high degree of accuracy in all models used, except for the models where an MVT of 0.40 m·s^−1^ was applied (Figure 1 and Figure 2) and for the models in females where an MVT of 0.30 was used. In these cases, the models consistently tended to underestimate the actual maximal values (Figure 2).

## 4. Discussion

The present study had several goals: First of all, to predict the 1RM in the free-weight back squat using the 1RM_MVT_ method; secondly, to analyze any differences in using two or three loads to predict the 1RM; and third, to evaluate, if any, differences in MVT between males and females and to assess how these differences might impact the accurate prediction of the 1RM in a female population. The results of this study highlight that the 1RM was predicted with remarkable accuracy using both two and three loads, using a MVT of 0.30 m·s^−1^ in males and 0.25 m·s^−1^ in females, and without any significant difference between the two models in both males and females. Furthermore, the models built with the optimal MVT of 0.40 m·s^−1^ revealed the worst accuracy among all the models (Figure 1 and Figure 2) for both males and females. Moreover, females showed a lower MVT than males. More importantly, this difference was crucial in accurately predicting the 1RM in females. Notably, the most inaccurate models were the 2-point and 3-point regression models for females using an MVT of 0.30 m·s^−1^, which is typically the MVT measured in males during the back squat, and the models built with the optimal MVT of 0.40 m·s^−1^. These models consistently underestimated the 1RM, had significantly higher SEE (Table 2) than the other models, and showed statistically significant differences between predicted and measured values (*p* < 0.05). These findings are partially in contrast with the scientific literature.

### 4.1. Importance of Sex-Specific Minimum Velocity Threshold

Consistent with previous studies, this study observed that females exhibit a lower MPV compared to males for light and intermediate loads [25,30,31]. However, contrary to the literature, a significant difference at higher (>80% 1RM) and maximal loads (MVT) was found [24,25,26]. Regarding MVT, a recent meta-analysis concluded that current data indicate no significant difference between males and females [24]. However, it is essential to note the limited amount of scientific evidence currently available on this topic, indicating that it remains an area requiring further investigation. Furthermore, the few existing studies that have analyzed the differences between males and females included males with greater training experience compared to the females [24]. This could partly explain why no significant differences in MPV were found at high or maximal loads since greater experience is known to negatively correlate with velocities at very high and maximal loads (MVT), with more experienced athletes showing lower velocities than novices or amateurs at the same load when it exceeds 85% 1RM [32]. Our results, on the other hand, highlighted significant differences even at maximal loads (MVT) when training experience was similar between sexes.

### 4.2. Importance of Load Selection for 1RM Prediction

Regarding the prediction of the 1RM using MVT in the back squat, our results are in contrast with the current literature. A recent systematic review and meta-analysis by LeMense et al. [10] highlighted how the current results do not allow for a precise estimation of the 1RM in the free-weight back squat using the 1RM_MVT_ method. Specifically, they reported that this methodology systematically overestimates the 1RM, with an error ranging from 3.4 to 20.1 kg. A possible explanation for these results, as hypothesized by Hughes et al. [21], is that the velocity measured at very light loads, even those above 20% 1RM and up to 35% 1RM, is less reliable due to the high velocities achieved during the execution. The poor reliability of the measured velocity may be attributed to the fact that high velocities have been associated with lower coordination and a greater variety of muscle activation patterns [33,34]. A recent study [15] used loads corresponding to 40% 1RM and 90% 1RM for the two points employed to develop the regression models, as previously suggested by Hughes et al. [21], and suggested the use of an “optimal MVT” of 0.40 m·s^−1^ to reduce the margin of error between predicted and measured values. Despite the existing evidence in the literature, our study revealed contrasting results. This may be explained by the numerous precautions taken during testing. First, as suggested by Hughes [21] et al. and later adopted by Chen et al. [15], the lightest load used for the regression model was approximately 35% 1RM, while the heaviest load remained below 90% 1RM, lower even than that used by Chen et al. [15]. Moreover, it is worth highlighting that the most accurate models were those developed for the female participants (Figure 2), where in fact, the highest load used was even lower, around 80% 1RM.

## 5. Conclusions

This finding suggests that future studies might benefit from investigating the use of maximal loads around 80% 1RM in male populations as well. Secondly, an MVT of 0.25 m·s^−1^, which represents the mean value for the tested females, was used to generate the regression models for females. Finally, throughout the entire incremental test, the researchers ensured that a correct and standardized execution technique was maintained at each load through the recording and evaluation of the lift by a certified FIPE referee. In conclusion, different MVT values should be used for males and females to achieve an accurate prediction of the 1RM. Furthermore, the use of an “optimal MVT” may be beneficial in contexts where accurately and precisely assessing lifting technique throughout the entire incremental test proves challenging. Conversely, when such assessment is feasible, our findings indicate that using average MVT values of 0.30 m·s^−1^ for males and 0.25 m·s^−1^ for females yield significantly more accurate results. However, to ensure accuracy, it is essential to use velocities that do not exceed 1 m·s^−1^ for the first point of the regression model and to maintain proper technical execution during each repetition.

## 6. Practical Application

Based on the results presented, it appears feasible to effectively predict the 1RM during the free-weight back squat using MVT, with loads ranging between 40% and 90% 1RM and using an MVT of 0.25 m·s^−1^ for females. It is important to emphasize that these results were obtained from highly trained subjects (1RM/BW ≥ 1.5). Furthermore, based on our results, the recommended number of loads to be used for creating regression models to predict the 1RM is two or three loads. Additionally, sex should be considered, as there are differences in MVT between males and females. Finally, the level of the athlete is crucial, as it has been demonstrated that athletes with a 1RM/BW > 1.5 ratio exhibit more accurate 1RM predictions using the load–velocity profile. Nevertheless, based on the strong validity, reliability, and accuracy demonstrated by our regression models, it is reasonable to suggest that using minimum loads close to 35% 1RM may allow these results to be replicated in less trained subjects with a 1RM/BW < 1.5. Based on these findings, further studies using the same protocol with less trained subjects, both males and females, are necessary to confirm the presented results.

## 7. Limitation

The main limitation of this study may lie in the values selected as MVTs for the female sample. While a threshold of 0.30 m·s^−1^ was used for the male participants, consistent with the previous literature and supported by several studies [20], the 0.25 m·s^−1^ MVT adopted for the female participants was determined based on the characteristics of our specific sample, as previously described. Therefore, future research should investigate how generalizable this value is to a similarly trained and experienced female population.

## Figures and Tables

**Table 1:** 1RM incremental test protocol [27].

Velocity (m·s^−1^)	Load Increment (kg)	Repetition	Rest (min)
	20	5	3
>0.8	20	3	3
0.6–0.8	10	2	3
0.5–0.6	5	1	3
<0.5	2.5	1	3

**Table 2:** Accuracy of the different regression models.

Model	SEE (kg) − (95%CI)	Pearson r	CV (%)
Males 2 points 0.4 m·s^−1^ MVT	10.09 − (−30.5; +11.49)	0.99	2.06
Males 3 points 0.4 m·s^−1^ MVT	9.86 − (−29.09; +10.87)	0.99	1.39
Males 2 points 0.3 m·s^−1^ MVT	2.86 − (−5.73; +5.85)	0.99	2.23
Males 3 points 0.3 m·s^−1^ MVT	1.98 − (−4.20; +3.96)	0.99	1.60
Females 2 points 0.4 m·s^−1^ MVT	12.61 − (−38.15; +13.93)	0.99	2.50
Females 3 points 0.4 m·s^−1^ MVT	12.62 − (−38.23; +13.89)	0.99	2.21
Females 2 points 0.3 m·s^−1^ MVT	4.20 − (−12.42; +4.88)	0.99	1.95
Females 3 points 0.3 m·s^−1^ MVT	4.26 − (−12.74; +4.80)	0.99	1.45
Females 2 points 0.25 m·s^−1^ MVT	1.76 − (−3.22; +4.04)	0.99	2.06
Females 3 points 0.25 m·s^−1^ MVT	1.21 − (−2.65; +2.63)	0.99	1.45

Table 2: SEE = standard error of estimate; CI = confidence intervals; CV = coefficient of variation; and MVT: minimum velocity threshold.

## Data Availability

Data available on request due to restriction (privacy).

## Abbreviations

The following abbreviations are used in this manuscript:

RT	Resistance Training
1RM	One-Repetition Maximum
MVT	Minimum Velocity Threshold
MPV	Mean Propulsive Velocity
SEE	Standard Error of Estimate
CV	Coefficient of Variation
